# Experiencing Dengue as a Medical Student

**DOI:** 10.31729/jnma.7915

**Published:** 2022-12-31

**Authors:** Shubham Shrestha

**Affiliations:** 1 Patan Academy of Health Sciences, Lagankhel, Lalitpur, Nepal

**Keywords:** *clopidogrel*, *dengue*, *medical student*

## Abstract

Dengue is a disease which spreads by the bite of an infected Aedes mosquito. Reading about a disease in a textbook and experiencing it as a patient is a completely different thing. This article highlights the feeling of a medical student as a patient. It provides the reader with an idea of how Basic Science knowledge can be used for rational decision-making. Self-prescription of medication without consulting a doctor can be more harmful in many circumstances. We also need to be aware of the warning signs of Dengue virus infection.

## INTRODUCTION

Dengue is a disease caused by Dengue virus. It spreads through the bite of an infected Aedes species mosquito. During the first week of infection, the dengue virus is found in the blood of an infected person. If a mosquito bites the infected person, the mosquito becomes infected. The infected mosquito can spread the virus to other people.^1^ The Dengue virus has four serotypes and it is possible to be infected four times. While many Dengue virus infections produce only mild illness, Dengue virus can cause an acute flu-like illness. Occasionally this develops into a potentially lethal complication, called severe dengue. If you have had dengue in the past, you are likely to develop severe dengue.^2^

In 2019, Nepal saw unexpected early rains which favored the dengue epidemic in 2019. This outbreak spread like wildfire reaching 68 out of 77 districts.^3^ Nepal experienced a surge in cases of Dengue in 2022 that started from the week commencing 8 August to 26 August. According to the Epidemiology and Disease Control Division (EDCD) of Ministry of Health and Population the highest new cases in 2022 have been reported in districts of Kathmandu, Laliptur and Makwanpur.^2^

## EXPERIENCE

The first time I was introduced with the term dengue was during an epidemic that occured in 2019. Having a travel history to Sunsari where there was Dengue prevalence followed by symptoms of headache and eye pain, I had been suspected of Dengue infection. But luckily it was not a Dengue infection.

As medical students, we often get asked by people about the common disease at any time. And at this time, it was Dengue in Kathmandu valley. It mostly would be easier and more fun work as a medical student to learn about the disease and answer people about their questions. Answering using a few medical terms learned so far can be a work of excitement to do. Saying people "Dengue is called break bone fever is an easy line", but when it came to facing it, it may a different story.

Dengue can be called as a synonym of pain. A pain that you might have never experienced before. You may have a high degree fever with nausea, vomiting, rash aches and pains (eye pain, typically behind the eyes, muscle, joint, or bone pain.^1^ With this pain, we may come to understand the term most often used in medical college so-called the importance of having empathy. Now as a patient, realization on how supportive it feels when someone shows empathy to you rather than sympathy having less meaning can be understood.

For a medical student who has just completed Basic Sciences and started Clinical Sciences, using learnt knowledge to interpret lab values can be a fun. But the thing that worries can be the severe dengue that may occur in Dengue fever. While learning about dengue from various online resources we come across severe outcomes of Dengue that might occur. Being cautious about the various warning signs like nasal bleeding, persistent vomiting, and severe abdominal pain is always required which if occurred then close monitoring is required and may be hospitalization.

For a patient infected with Dengue supportive treatment is recommended as Dengue is a viral infection and antibiotics aren't effective in viral illness. Viral infection causes decrease in white blood cell count cell count. White Blood cells are the part of immune system that protects your body from infection. So with decrease in White blood cells patient may suffer from infections. After few days of relief from headache and myalgia, I started having tonsillitis and pharyngitis. After few doses of antibiotics for it, I started having antibiotic associated diarrhoea. It was a situation where I was unable to take any food due to pharyngitis and was having lots of fluid loss due to diarrhoea. With stopping of the antibiotics for certain duration and timely intake of ORS solution helped tackle this situation.

## CLOPIDOGREL AND DENGUE INFECTION

In dengue, the pathophysiology of thrombocytopenia is not clearly understood. It is believed that it rests mainly on two events: decreased bone marrow production and increased peripheral destruction and clearance of platelets.^4^ Clopidogrel is an inhibitor of platelet activation and aggregation through the irreversible binding of its active metabolite to the P2Y12 class of ADP receptors on platelets.^5^ Using this Basic Sciences knowledge learnt so far, I recommended my mother stop Clopidogrel as her regular medicine during her dengue infection.

## NON-STEROIDAL ANTI-INFLAMMATORY AGENTS (NSAIDS) AND DENGUE INFECTION

Having knowledge of Pharmacology that NSAIDS which are generally used as analgesics also have the action of inhibiting platelet aggregation and secretion by inhibiting cyclooxygenase helped me avoid nonsteroidal anti-inflammatory agents (NSAIDS) during dengue infection.

Consensus guidelines for the treatment of dengue fever from the World Health Organization recommend acetaminophen to manage pain and fever but contraindicate non-steroidal anti-inflammatory agents (NSAIDS) because of potentially increased bleeding risk, with thrombocytopenia as a complication.^6^

## MEDICINE INTAKE WITHOUT DOCTOR CONSULTATION

Self-medication can be defined as obtaining and consuming drugs without the advice of a physician either for diagnosis, prescription or surveillance of treatment.^6^ Fever and headache are the most common reasons for a non-doctor prescription.^7^ In many mild health issues patients tend to prefer self-medication.

The pharmacist plays an important role in health care by selling the prescribed medicine by doctors and helping in the overall treatment of the patient's disease. In many cases, we can see that many people have the tendency to go to a pharmacy and ask for the medication they have been prescribed by themselves and start taking it even without a proper dosing schedule. To some extent, these practices can solve the patient's problem with minor illnesses. But this can be considered a problem in medical practices if the following situation exists. Doctors aim to treat patients and get them to recover. But activities here like random self-prescription may sometime prove to be harmful to the patient.

There can be a situation where a patient with body aches and headaches goes to the pharmacy and asks for analgesics as the first choice and it probably may be NSAIDs. The patient buys it and starts taking it. His/her pain may subside but the question is, isn't this practice worsening the Disease status in real if it was a dengue infection? Who here is considered responsible for an adverse decrease in platelet count that might occur in dengue patients taking NSAIDs? The patient now lands up with a severe decrease in platelet count. The story would have been different if the patient would have consulted the doctor at that very instant. She/he would have been diagnosed with dengue and would be aware of such Does and Don'ts by the doctors.

## WAY FORWARD

Dengue is a disease which can be prevented by simple actions like using mosquito repellents. As dengue infection depletes platelets so NSAIDs and anti-platelet drugs like Clopidogrel should be avoided during this period. Awareness programs from Mass communication media should also include the things that should be avoided during disease conditions. We need to be aware of the warning signs of dengue virus infection. Education to help patients decide on the appropriateness of self-medication is required in developing countries like Nepal.

## References

[1] Centres for Disease Control and Prevention. (2021). Dengue [Internet]..

[2] World Health Organization. (10). Dengue and Severe Dengue [Internet]..

[3] Pandey BD, Costello A (2019). The dengue epidemic and climate change in Nepal.. Lancet..

[4] Boo YL, Lim SY, Png HS, Liam C, Huan NC (2019). Persistent thrombocytopenia following dengue fever: What should we do?. Malays Fam Physician..

[5] Niitsu Y, Sugidachi A, Ogawa T, Jakubowski JA, Hashimoto M, Isobe T (2008). Repeat oral dosing of prasugrel, a novel P2Y12 receptor inhibitor, results in cumulative and potent antiplatelet and antithrombotic activity in several animal species.. Eur J Pharmacol..

[6] Kellstein D, Fernandes L (2019). Symptomatic treatment of dengue: should the NSAID contraindication be reconsidered?. Postgrad Med..

[7] Montastruc JL, Bagheri H, Geraud T, Lapeyre-Mestre M. (1997). cc Therapie..

[8] Shankar PR, Partha P, Shenoy N (2002). Self-medication and non-doctor prescription practices in Pokhara valley, Western Nepal: a questionnaire-based study.. BMC Fam Pract.

